# On Poisson Nonlinear Transformations

**DOI:** 10.1155/2014/832861

**Published:** 2014-07-17

**Authors:** Nasir Ganikhodjaev, Nur Zatul Akmar Hamzah

**Affiliations:** Department of Computational and Theoretical Sciences, Faculty of Science, International Islamic University Malaysia, 25710 Kuantan, Malaysia

## Abstract

We construct the family of Poisson nonlinear transformations defined on the countable sample space of nonnegative integers and investigate their trajectory behavior. We have proved that these nonlinear transformations are regular.

## 1. Introduction

Let (*X*, *F*) be a measurable space, where *X* is a state space and *F* is *σ*-algebra on *X*, and *S*(*X*, *F*) the set of all probability measures on (*X*, *F*).

Let {*P*(*x*, *y*, *A*) : *x*, *y* ∈ *X*, *A* ∈ *F*} be a family of functions on *X* × *X* × *F* such that, for any fixed *x*, *y* ∈ *X* *P*(*x*, *y*, ·) ∈ *S*(*X*, *F*), *P*(*x*, *y*, *A*) regarded as a function of two variables *x* and *y* with fixed *A* ∈ *F* is a measurable function on (*X* × *X*, *F* ⊗ *F*) and *P*(*x*, *y*, *A*) = *P*(*y*, *x*, *A*) for any *x*, *y* ∈ *X* and *A* ∈ *F*.

We consider a nonlinear transformation called quadratic stochastic operator (qso) *V* : *S*(*X*, *F*) → *S*(*X*, *F*) which is defined by

(1) $$\begin{array}{c} \left(V\lambda \right)\left(A\right)=\overset{}{\underset{X}{\iint }}P\left(x,y,A\right)d\lambda \left(x\right)d\lambda \left(y\right), \end{array}$$

where *A* ∈ *F* is an arbitrary measurable set.

If a state space *X* = {1,2,…, *m*} is a finite set and the corresponding *σ*-algebra is the power set *P*(*X*), that is, the set of all subsets of *X*, then the set of all probability measures on (*X*, *F*) has the following form:

(2) Sm−1={x=(x1,x2,…,xm)∈Rm:xi≥0     for  any  i,  and  ∑i=1mxi=1}

that is called a (*m* − 1)-dimensional simplex.

In this case, the probabilistic measure *P*(*i*, *j*, ·) is a discrete measure with ∑_*k*=1_^*m*^ *P*(*ij*, {*k*}) = 1, where *P*(*ij*, {*k*}) ≡ *P*_*ij*,*k*_ for any *i*, *j* ∈ *X*. In addition, the corresponding qso V has the following form:

(3) $$\begin{array}{c} {\left(Vx\right)}_{k}=\overset{m}{\underset{i,j=1}{\sum }}P_{ij,k}x_{i}x_{j}, \end{array}$$

for any *x* ∈ *S* ^*m*−1^ and the coefficients *P*_*ij*,*k*_ satisfy the following conditions:

(4) (a)  Pij,k≥0;(b)  Pij,k=Pji,k;(c)  ∑k=1mPij,k=1 ∀ i,j,k∈{1,2,…,m}.

Such operator can be reinterpreted in terms of evolutionary operator of free population [1–10] and in this form it has a fair history.

In this paper, we consider nonlinear transformations defined on countable state space and investigate their limit behavior of trajectories.

## 2. A Poisson qso

Let *X* = {0,1,…} be a countable sample space and corresponding *σ*-algebra *F* a power set *P*(*X*), that is, the set of all subsets of *X*. In order to define a probability measure *μ* on countable sample space *X*, it is enough to define the measure *μ*({*k*}) of each singleton {*k*}, *k* = 0,1,…. Thus, we will write *μ*(*k*) instead of *μ*({*k*}).

Let {*P*(*i*, *j*, *k*) : *i*, *j*, *k* ∈ *X*} be a family of functions defined on *X* × *X* × *F*, which satisfy the following conditions:

- *P*(*i*, *j*, ·) is a probability measure on (*X*, *F*) for any fixed *i*, *j* ∈ *X*;
- *P*(*i*, *j*, *k*) = *P*(*j*, *i*, *k*) ≡ *P*_*ij*,*k*_, where *k* ∈ *X* for any fixed *i*, *j* ∈ *X*.

In this case, a qso (1) on measurable space (*X*, *F*) is defined as follows:

(5) $$\begin{array}{c} V\mu \left(k\right)=\overset{\infty }{\underset{i=0}{\sum }}\overset{\infty }{\underset{j=0}{\sum }}P_{ij,k}\mu \left(i\right)\mu \left(j\right), \end{array}$$

where *k* ∈ *X* for arbitrary measure *μ* ∈ *S*(*X*, *F*).

In this paper, we consider a Poisson qso which is a Poisson distribution *P*_*λ*_ with a positive real parameter *λ* defined on *X* by the equation

(6) $$\begin{array}{c} P_{\lambda }\left(k\right)=e^{-\lambda }\frac{\lambda^{k}}{k!}, \end{array}$$

for any *k* ∈ *X*.

Let *S*(*X*, *F*) be a set of all probability measures on (*X*, *F*) and let *P*(*i*, *j*, ·) be a probability measure on (*X*, *F*) for any *i*, *j* ∈ *X*.

**Definition 1 .**

A quadratic stochastic operator *V* (5) is called a Poisson qso if, for any *i*, *j* ∈ *X*, the probability measure *P*(*i*, *j*, ·) is the Poisson distribution *P*_*λ*(*i*,*j*)_ with positive real parameters *λ*(*i*, *j*), where *λ*(*i*, *j*) = *λ*(*j*, *i*).

Assume that {*V* ^*n*^ *λ* : *n* = 0,1, 2,…} is the trajectory of the initial point *λ* ∈ *S*(*X*, *F*), where *V* ^*n*+1^ *λ* = *V*(*V* ^*n*^ *λ*) for all *n* = 0,1, 2,…, with *V* ^0^ *λ* = *λ*.

In this paper, we will study limit behavior of trajectories of Poisson qso.

## 3. Ergodicity and Regularity of qso

Let us consider a qso *V* (5) defined on countable set *X*. Let {*V* ^*n*^ *λ* : *n* = 0,1, 2,…} be the trajectory of the initial point *λ* ∈ *S*(*X*, *F*), where *V* ^*n*+1^ *λ* = *V*(*V* ^*n*^ *λ*) for all *n* = 0,1, 2,….

**Definition 2 .**

A measure *μ* ∈ *S*(*X*, *F*) is called a fixed point of a qso *V* if *Vμ* = *μ*.

Let Fix⁡(*V*) be the set of all fixed points of qso *V*.

**Definition 3 .**

A qso *V* is called regular if, for any initial point *μ* ∈ *S*(*X*, *F*), the limit

(7) $$\begin{array}{c} \underset{n\to \infty }{\lim}V^{n}\left(\mu \right) \end{array}$$

exists.

In measure theory, there are various notions of the convergence of measures: weak convergence, strong convergence, and total variation convergence. Below we consider strong convergence.

**Definition 4 .**

For (*X*, *F*) a measurable space, a sequence *μ*_*n*_ is said to converge strongly to a limit *μ* if

(8) $$\begin{array}{c} \underset{n\to \infty }{\lim}\mu_{n}\left(A\right)=\mu \left(A\right), \end{array}$$

for every set *A* ∈ *F*.

If *X* is a countable set, then a sequence *μ*_*n*_ converges strongly to a limit *μ* if and only if

(9) $$\begin{array}{c} \underset{n\to \infty }{\lim}\mu_{n}\left(k\right)=\mu \left(k\right), \end{array}$$

for every singleton *k* ∈ *X*.

In statistical mechanics, the ergodic hypothesis proposes a connection between dynamics and statistics. In the classical theory, the assumption was made that the average time spent in any region of phase space is proportional to the volume of the region in terms of the invariant measure. More generally, such time averages may be replaced by space averages.

For nonlinear dynamical systems, Ulam [11] suggested an analogous measure-theoretic ergodicity with following ergodic hypothesis.

**Definition 5 .**

A nonlinear operator *V* defined on *S*(*X*, *F*) is called ergodic, if the limit

(10) $$\begin{array}{c} \underset{n\to \infty }{\lim}\frac{1}{n}\overset{n-1}{\underset{k=0}{\sum }}V^{k}\lambda \end{array}$$

exists for any *λ* ∈ *S*(*X*, *F*).

On the ground of numerical calculations for quadratic stochastic operators defined on *S*(*X*, *F*) with finite *X*, Ulam [11] conjectured that the ergodic theorem holds for any such qso *V*.

In 1977, Zakharevich [12] proved that this conjecture is false in general. He considered the following operator on *S* ^2^:

(11) x1′=x12+2x1x2,x2′=x22+2x2x3,x3′=x32+2x1x3,

and he proved that such operator is nonergodic transformation. Later in [13], the sufficient condition to be nonergodic transformation was established for qso defined on *S* ^2^.

In the next section, we will show that Ulam's conjecture is true for some class of Poisson qso.

## 4. Ergodicity and Regularity of Poisson qso

Let *V* defined in (5) be a Poisson qso. We consider the following cases.

### 4.1. Homogenious Poisson qso

We call a Poisson quadratic stochastic operator *V* (5) homogenious, if *λ*(*i*, *j*) = *λ*, for any *i*, *j* ∈ *X*, that is, *P*_*ij*,*k*_ = *e* ^−*λ*^(*λ* ^*k*^/*k*!). Then for arbitrary measure *μ* ∈ *S*(*X*, *F*)

(12) $$\begin{array}{c} V\mu \left(k\right)=\overset{\infty }{\underset{i=0\;}{\sum }}\overset{\infty }{\underset{j=0}{\sum }}P_{ij,k}\mu \left(i\right)\mu \left(j\right)=e^{-\lambda }\frac{\lambda^{k}}{k!}, \end{array}$$

where *k* ∈ *X*, that is, *Vμ* = *P*_*λ*_.

Thus *V* ^*n*^ *μ* = *P*_*λ*_ for any *n* = 1,2,…, that is, Fix⁡(*V*) = *P*_*λ*_, and we have the following statement.

**Proposition 6 .**

A homogenious Poisson qso is a regular transformation.

### 4.2. Poisson qso with Two Different Parameters

We consider a Poisson qso such that

(13) $$\begin{array}{c} P_{ij,k}=\{\begin{array}{cc} e^{-\lambda_{1}}\frac{\lambda_{1}^{k}}{k!} & \text{if}\;i+j\;\;\text{is}\;\text{even},\\ e^{-\lambda_{2}}\frac{\lambda_{2}^{k}}{k!} & \text{if}\;i+j\;\text{is}\;\text{odd}. \end{array} \end{array}$$

For any initial measure *μ* ∈ *S*(*X*, *F*) let

(14) $$\begin{array}{c} A\left(\mu \right)=\overset{\infty }{\underset{n=0}{\sum }}\mu \left(2n\right),\;\;B\left(\mu \right)=\overset{\infty }{\underset{n=0}{\sum }}\mu \left(2n+1\right), \end{array}$$

where *A*(*μ*) + *B*(*μ*) = 1. It is easy to show that for Poisson distribution *P*_*λ*_

(15) $$\begin{array}{c} A\left(P_{\lambda }\right)=\frac{1+e^{-2\lambda }}{2},\;\;B\left(P_{\lambda }\right)=\frac{1-e^{-2\lambda }}{2}. \end{array}$$

Then for any measure *μ* ∈ *S*(*X*, *F*), we have

(16) Vμ(k)=∑i=0 ∞∑j=0∞Pij,kμ(i)μ(j)=∑m,n=0∞[P2m,2n,kμ(2m)μ(2n)   +P2m+1,2n+1,kμ(2m+1)μ(2n+1)]+∑m,n=0∞[P2m+1,2n,kμ(2m+1)μ(2n)     +P2m,2n+1,kμ(2m)μ(2n+1)]=e−λ1λ1kk![A2(μ)+B2(μ)]+e−λ2λ2kk![2A(μ)B(μ)],V2μ(k)=∑i=0 ∞∑j=0∞Pij,kVμ(i)Vμ(j)=∑m,n=0∞[P2m,2n,kVμ(2m)Vμ(2n)   +P2m+1,2n+1,kVμ(2m+1)Vμ(2n+1)]+∑m,n=0∞[P2m+1,2n,kVμ(2m+1)Vμ(2n)    +P2m,2n+1,kVμ(2m)Vμ(2n+1)]=e−λ1λ1kk![A2(Vμ)+B2(Vμ)]+e−λ2λ2kk![2A(Vμ)B(Vμ)].

By simple calculations, we have

(17) A(Vμ)=1+e−2λ12[A2(μ)+B2(μ)]+1+e−2λ22[2A(μ)B(μ)], B(Vμ)=1−e−2λ12[A2(μ)+B2(μ)]+1−e−2λ22[2A(μ)B(μ)].

Thus, by using induction on the sequence *V* ^*n*^(*μ*), we produce the following recurrent equation:

(18) Vn+1μ(k)=e−λ1λ1kk![A2(Vnμ)+B2(Vnμ)]+e−λ2λ2kk![2A(Vnμ)B(Vnμ)],

where *n* = 0,1,…, Besides, for parameters *A*(*V* ^*n*^ *μ*) and *B*(*V* ^*n*^ *μ*), we have the following recurrent equations:

(19) A(Vn+1μ)=1+e−2λ12[A2(Vnμ)+B2(Vnμ)]+1+e−2λ22[2A(Vnμ)B(Vnμ)],B(Vn+1μ)=1−e−2λ12[A2(Vnμ)+B2(Vnμ)]+1−e−2λ22[2A(Vnμ)B(Vnμ)].

It is obvious that the limit behavior of the recurrent equation (18) is fully determined by limit behavior of recurrent equations (19).

Since *A*(*V* ^*n*^ *μ*) + *B*(*V* ^*n*^ *μ*) = 1, where *A*(*V* ^*n*^ *μ*) ≥ 0 and *B*(*V* ^*n*^ *μ*) ≥ 0, the recurrent equations (19) are rewritten as follows:

(20) x′=A(λ1)(x2+y2)+2A(λ2)xy,y′=B(λ1)(x2+y2)+2B(λ2)xy

with *x* ≥ 0, *y* ≥ 0, and *x* + *y* = 1.

**Figure 1:**
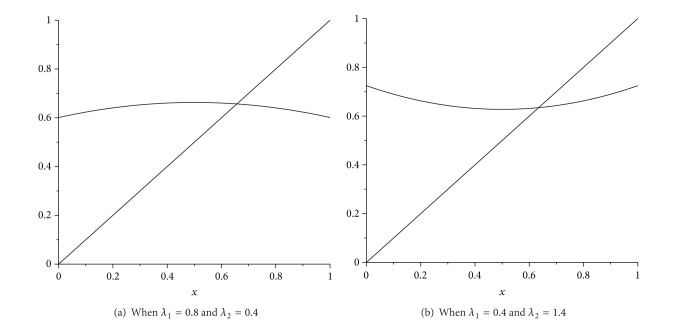
Graph of the function (21) for some fixed values *λ*_1_ and *λ*_2_.

Solving the following quadratic equation

(21) $$\begin{array}{c} x=A\left(\lambda_{1}\right)\left(x^{2}+{\left(1-x\right)}^{2}\right)+2A\left(\lambda_{2}\right)x\left(1-x\right), \end{array}$$

we have single fixed point and denoted it as (x*, y*) (see Figure 1). Using simple calculus (see Figure 1), one can show that any trajectory of the qso (20) defined on one-dimensional simplex *S* ^1^ converges to this fixed point; that is, qso (20) is regular transformation, so that it is ergodic.

Thus, for any initial measure *μ*, we have

(22) $$\begin{array}{c} \underset{n\to \infty }{\lim}A\left(V^{n}\mu \right)=x^{*},\;\;\underset{n\to \infty }{\lim}B\left(V^{n}\mu \right)=y^{*}. \end{array}$$

Then, passing to limit in (18), for any singleton *k*, we have

(23) lim⁡n→∞Vn+1μ(k) =lim⁡n→∞{e−λ1λ1kk![A2(Vnμ)+B2(Vnμ)]      +e−λ2λ2kk![2A(Vnμ)B(Vnμ)]} =e−λ1λ1kk![x∗2+y∗2]+e−λ2λ2kk![2x∗y∗] =[x∗2+y∗2]Pλ1(k)+[2x∗y∗]Pλ2(k).

Thus, for any initial measure *μ*, the strong limit of the sequence *V* ^*n*^ *μ* exists and is equal to the convex linear combination

(24) $$\begin{array}{c} \underset{n\to \infty }{\lim}V^{n}\mu \left(k\right)=\left({x^{*}}^{2}+{y^{*}}^{2}\right)P_{\lambda_{1}}\left(k\right)+2x^{*}y^{*}P_{\lambda_{2}}\left(k\right), \end{array}$$

of two Poisson measures *P*_*λ*_1__ and *P*_*λ*_2__. It is evident that Fix⁡(*V*) = (x*^2^ + y*^2^)*P*_*λ*_1__(*k*) + 2x*y*P_*λ*_2__(*k*).

As corollary we have following statement.

**Proposition 7 .**

A Poisson qso with two different parameters is a regular and, respectively, ergodic transformation with respect to strong convergence.

### 4.3. A Poisson qso with Three Different Parameters

We consider a Poisson qso such that

(25) $$\begin{array}{c} P_{ij,k}=\{\begin{array}{cc} e^{-\lambda_{1}}\frac{\lambda_{1}^{k}}{k!} & \text{if}\;\;i+j=0\left(\mathrm{mod}\;3\right),\\ e^{-\lambda_{2}}\frac{\lambda_{2}^{k}}{k!} & \text{if}\;\;i+j=1\left(\mathrm{mod}\;3\right),\\ e^{-\lambda_{3}}\frac{\lambda_{3}^{k}}{k!} & \text{if}\;\;i+j=2\left(\mathrm{mod}\;3\right). \end{array} \end{array}$$

For any initial measure *μ* ∈ *S*(*X*, *F*), let

(26) $$\begin{array}{c} A\left(\mu \right)=\overset{\infty }{\underset{n=0}{\sum }}\mu \left(3n\right),\;\;B\left(\mu \right)=\overset{\infty }{\underset{n=0}{\sum }}\mu \left(3n+1\right),\\ C\left(\mu \right)=\overset{\infty }{\underset{n=0}{\sum }}\mu \left(3n+2\right), \end{array}$$

where *A*(*μ*) + *B*(*μ*) + *C*(*μ*) = 1. It is easy to show that, for Poisson distribution *P*_*λ*_ with parameter *λ*, we have

(27) A(λ)=1+2e−(3/2)λcos⁡(3/2)λ3,B(λ)=1−2e−(3/2)λcos⁡⁡((3/2)λ+π/3)3,C(λ)=1−2e−(3/2)λcos⁡((3/2)λ−π/3)3.

Then, for any measure *μ* ∈ *S*(*X*, *F*), we have

(28) Vμ(k)=∑i=0 ∞∑j=0∞Pij,kμ(i)μ(j)=∑m,n=0∞[P3m,3n,kμ(3m)μ(3n)   +P3m+1,3n+2,kμ(3m+1)μ(3n+2)   +P3m+2,3n+1,kμ(3m+2)μ(3n+1)]+∑m,n=0∞[P3m+1,3n,kμ(3m+1)μ(3n)    +P3m,3n+1,kμ(3m)μ(3n+1)    +P3m+2,3n+1,kμ(3m+2)μ(3n+1)]+∑m,n=0∞[P3m+2,3n,kμ(3m+2)μ(3n)    +P3m,3n+2,kμ(3m)μ(3n+2)    +P3m+1,3n+1,kμ(3m+1)μ(3n+1)]=e−λ1λ1kk![A2(μ)+2B(μ)C(μ)]+e−λ2λ2kk![2A(μ)B(μ)+C2(μ)]+e−λ3λ3kk![2A(μ)C(μ)+B2(μ)],V2μ(k)=∑i=0∞∑j=0∞Pij,kVμ(i)Vμ(j)=∑m,n=0∞[P3m,3n,kVμ(3m)Vμ(3n)   +P3m+1,3n+2,kVμ(3m+1)Vμ(3n+2)   +P3m+2,3n+1,kVμ(3m+2)Vμ(3n+1)]+∑m,n=0∞[P3m+1,3n,kVμ(3m+1)Vμ(3n)    +P3m,3n+1,kVμ(3m)Vμ(3n+1)    +P3m+2,3n+2,kVμ(3m+2)Vμ(3n+2)]+∑m,n=0∞[P3m+2,3n,kVμ(3m+2)Vμ(3n)    +P3m,3n+2,kVμ(3m)Vμ(3n+2)    +P3m+1,3n+1,kVμ(3m+1)Vμ(3n+1)]=e−λ1λ1kk![A2(Vμ)+B(Vμ)C(Vμ)]+e−λ2λ2kk![2A(Vμ)B(Vμ)+C2(Vμ)]+e−λ3λ3kk![2A(Vμ)C(Vμ)+B2(Vμ)].

By simple calculations, we have

(29) A(Vμ)=A(λ1)[A2(μ)+2B(μ)C(μ)]+A(λ2)[2A(μ)B(μ)+C2(μ)]+A(λ3)[2A(μ)C(μ)+B2(μ)],B(Vμ)=B(λ1)[A2(μ)+2B(μ)C(μ)]+B(λ2)[2A(μ)B(μ)+C2(μ)]+B(λ3)[2A(μ)C(μ)+B2(μ)],C(Vμ)=C(λ1)[A2(μ)+2B(μ)C(μ)]+C(λ2)[2A(μ)B(μ)+C2(μ)]+C(λ3)[2A(μ)C(μ)+B2(μ)].

Thus, by using induction on sequence *V* ^*n*^(*μ*), we produce the following recurrent equation:

(30) Vn+1μ(k)=e−λ1λ1kk![A2(Vnμ)+2B(Vnμ)C(Vnμ)]+e−λ2λ2kk![2A(Vnμ)B(Vnμ)+C2(Vnμ)]+e−λ3λ3kk![2A(Vnμ)C(Vnμ)+B2(Vnμ)],

where *n* = 0,1,…. Besides, for parameters *A*(*V* ^*n*^ *μ*), *B*(*V* ^*n*^ *μ*) and *C*(*V* ^*n*^ *μ*), we have the following recurrent equations:

(31) A(Vn+1μ)=A(λ1)[A2(Vnμ)+2B(Vnμ)C(Vnμ)]+A(λ2)[2A(Vnμ)B(Vnμ)+C2(Vnμ)]+A(λ3)[2A(Vnμ)C(Vnμ)+B2(Vnμ)],B(Vn+1μ)=B(λ1)[A2(Vnμ)+2B(Vnμ)C(Vnμ)]+B(λ2)[2A(Vnμ)B(Vnμ)+C2(Vnμ)]+B(λ3)[2A(Vnμ)C(Vnμ)+B2(Vnμ)],C(Vn+1μ)=C(λ1)[A2(Vnμ)+2B(Vnμ)C(Vnμ)]+C(λ2)[2A(Vnμ)B(Vnμ)+C2(Vnμ)]+C(λ3)[2A(Vnμ)C(Vnμ)+B2(Vnμ)].

It is obvious that the limit behavior of the recurrent equation (30) is fully determined by limit behavior of recurrent equations (31).

Since *A*(*V* ^*n*^ *μ*) + *B*(*V* ^*n*^ *μ*) + *C*(*V* ^*n*^ *μ*) = 1, where *A*(*V* ^*n*^ *μ*) ≥ 0, *B*(*V* ^*n*^ *μ*) ≥ 0, and *C*(*V* ^*n*^ *μ*) ≥ 0, the recurrent equations (31) are rewritten as follows:

(32) x′=A(λ1)x2+A(λ3)y2+A(λ2)z2+2A(λ2)xy+A(λ3)xz+A(λ1)yz,y′=B(λ1)x2+B(λ3)y2+B(λ2)z2+2B(λ2)xy+B(λ3)xz+B(λ1)yz,z′=C(λ1)x2+C(λ3)y2+C(λ2)z2+2C(λ2)xy+C(λ3)xz+C(λ1)yz,

where *x* + *y* + *z* = 1.

**Figure 2:**
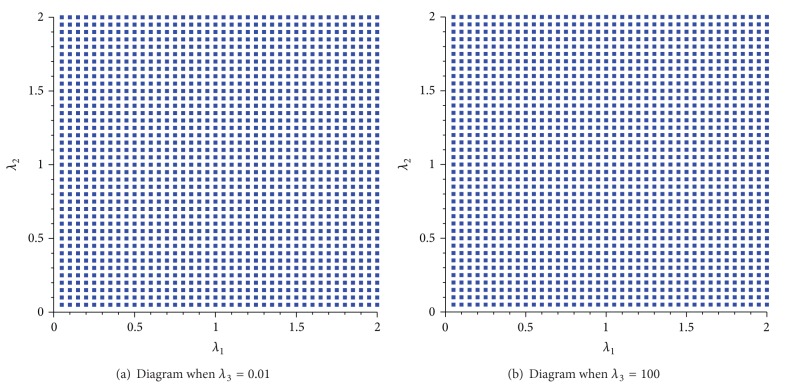
Limit behavior of the dynamical system (31) 0 < *λ*_1_, *λ*_2_ ≤ 2 and some fixed values *λ*_3_.

Starting from arbitrary initial data, we iterate the recurrence equations (31) and observe their behavior after a large number of iterations. The resultant diagram in the space (*λ*_1_, *λ*_2_) with 0 < *λ*_1_, *λ*_2_ ≤ 2, and some fixed *λ*_3_ are shown in Figure 2. In this diagram, blue color corresponds to the converges of the trajectory.

One can prove that for any values of parameters *λ*_1_, *λ*_2_, and *λ*_3_ the nonlinear transformation (31) has a single fixed point (x*, y*, z*) and, respectively, it is regular transformation.

If these parameters are very small, for instance, *λ*_1_ = 3 · 10^−15^, *λ*_2_ = 2 · 10^−15^, and *λ*_3_ = 1 · 10^−15^, then any trajectory converges to (1,0, 0). But, if they are rather large, for instance, *λ*_1_ = 25, *λ*_2_ = 50, and *λ*_3_ = 75, then any trajectory converges to (1/3,1/3,1/3).

As above, from (31) it follows that for any singleton *k* ∈ *X* the limit of the sequence *V* ^*n*^ *μ*(*k*) exists and equals

(33) lim⁡n→∞Vn+1μ(k)=e−λ1λ1kk![x∗2+2y∗z∗]+e−λ2λ2kk![2x∗y∗+z∗2]+e−λ3λ3kk![2x∗z∗+y∗2].

Thus, the strong limit of the sequence *V* ^*n*^ *μ* exists and equals convex linear combination

(34) lim⁡n→∞Vn+1μ=(x∗2+2y∗z∗)Pλ1+(2x∗y∗+z∗2)Pλ2+(2x∗z∗+y∗2)Pλ3,

of three Poisson measures *P*_*λ*_1__, *P*_*λ*_2__, and *P*_*λ*_3__. It is evident that Fix⁡(*V*) = (x*^2^ + 2y*z*)*P*_*λ*_1__ + (2x*y* + z*^2^)*P*_*λ*_2__ + (2x*z* + y*^2^)*P*_*λ*_3__.

As corollary we have following statement.

**Proposition 8 .**

A Poisson qso with three different parameters is a regular and, respectively, ergodic transformation with respect to strong convergence.

## 5. Conclusion

In this paper, we present a construction of Poisson quadratic stochastic operators and prove their regularity when the number of different parameters *λ*_*i*_ is less than or equal to three. The Poisson quadratic stochastic operators with any finitely many different parameters *λ*_*i*_ and countably many different parameters *λ*_*i*_ will be considered in another paper.
